# Emerging Roles for Immune Cells and MicroRNAs in Modulating the Response to Cardiac Injury

**DOI:** 10.3390/jcdd6010005

**Published:** 2019-01-15

**Authors:** Adriana M. Rodriguez, Viravuth P. Yin

**Affiliations:** Kathryn W. Davis Center for Regenerative Biology and Medicine, MDI Biological Laboratory, Salisbury Cove, ME 04672, USA; arodriguez@mdibl.org

**Keywords:** myocardial infarction, heart regeneration, macrophages, regulatory T cells, microRNAs, zebrafish, cardiomyocyte proliferation, fibrosis

## Abstract

Stimulating cardiomyocyte regeneration after an acute injury remains the central goal in cardiovascular regenerative biology. While adult mammals respond to cardiac damage with deposition of rigid scar tissue, adult zebrafish and salamander unleash a regenerative program that culminates in new cardiomyocyte formation, resolution of scar tissue, and recovery of heart function. Recent studies have shown that immune cells are key to regulating pro-inflammatory and pro-regenerative signals that shift the injury microenvironment toward regeneration. Defining the genetic regulators that control the dynamic interplay between immune cells and injured cardiac tissue is crucial to decoding the endogenous mechanism of heart regeneration. In this review, we discuss our current understanding of the extent that macrophage and regulatory T cells influence cardiomyocyte proliferation and how microRNAs (miRNAs) regulate their activity in the injured heart.

## 1. Introduction

Cardiovascular disease is the most prevalent cause of death in the USA and in the Western world. Coronary heart disease (CHD) is the most common type of heart disease and is responsible for ~60% of those deaths [1]. A study based on 2010 figures estimates that by 2040, the prevalence of CHD in the U.S. will have increased by 47%, from 11.7 million to 17.3 million affected individuals, and associated annual health care costs will rise 41%, from $126.2 billion to $177.5 billion [2,3].

CHD develops in response to accumulations of calcium phosphate- and lipid-containing plaques in blood vessels, which restricts oxygenated blood flow to heart muscle. Myocardial infarction (MI) or a heart attack is due to loss of blood flow that causes local ischemia and subsequent death of the cardiomyocytes (CMs) through necrosis and apoptosis. In adult mammals, ischemic injury is repaired through the formation of non-contractile scar tissue, which increases the burden on the remaining heart muscle, frequently leading to heart failure and death [4].

While standard treatments, such as reperfusions [5], help mitigate cardiac damage post-MI, they merely stabilize the heart at its current damaged-state by protecting viable cells in the area at risk (or peri-infarct zone) from ischemia-induced death. Thus, to truly improve cardiac function in MI patients—without necessitating a rare heart transplant [6]—a concerted effort should be directed toward advancing strategies that help restore its contractile function. To meet this need, scientists in regenerative biology have been studying how animals like the zebrafish and axolotl, which have a remarkable endogenous ability to regenerate their tissues and organs, are able to structurally and functionally recover after cardiac injury [7,8,9,10].

In response to major injury such as resection of the ventricular apex, surface cryoinjury, or genetic ablation of up to 60% of CMs, the zebrafish heart initiates repair with deposition of temporary fibrin and collagen tissue [7,11,12,13,14,15,16]. Within the first 2 weeks after injury, spared CMs dedifferentiate and proliferate, and collagen scar tissue is degraded, thus resulting in new muscle formation and restoration of lost heart function [17,18]. Over the past decade, much progress has been made in understanding the sequence of cellular events that lead from ischemic injury to recovery in the zebrafish heart as well as molecular factors required for the process to proceed towards a pro-regenerative course [19]. Given that at least 20 signaling factors have already been shown to be required for CM proliferation alone, an effective regenerative cocktail is likely one that can target multiple factors. Two such multi-targeted injury-response modulators have recently emerged as promising therapeutic targets: (1) immune cells [20,21,22,23,24,25,26] and (2) microRNAs (miRNAs) [27,28,29,30].

This review focuses on the cardiac-injury response, highlighting variations that arise between pro-fibrotic and pro-regenerative responses, followed by a discussion on how these responses are naturally modulated by the immune cells and microRNAs in infarcted hearts.

## 2. Pro-Fibrotic and Pro-Regenerative Deviations in the Response to Cardiac Injury

**Figure 1:**
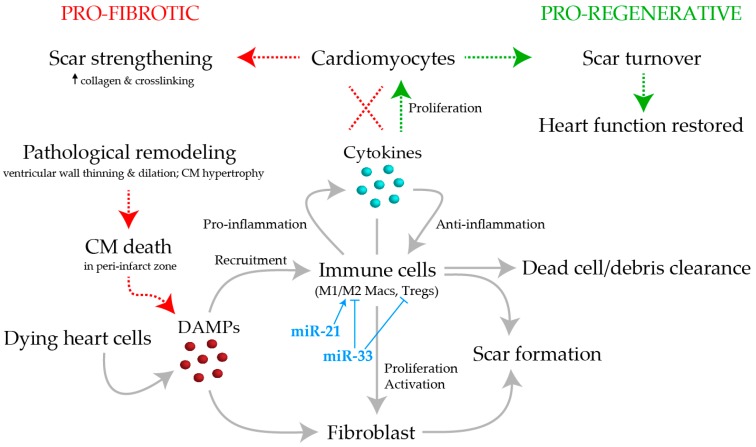
Schematic overview of the core cardiac repair program. The pro-fibrosis and pro-regenerative cascades are separate arms of a unified core program. Arrows indicate events associated with a pro-fibrotic response (dashed, red) or a pro-regenerative response (dashed, green). Dashed “X” indicates failure to induce cardiomyocyte (CM) proliferation. DAMPs = damage-associated molecular patterns; Macs = macrophages; Tregs = regulatory T cells.

While pro-fibrotic and pro-regenerative responses to cardiac injury are often envisioned as two entirely separate programs, here we will describe them as deviations of a core cardiac repair program (Figure 1). The core program is initiated with the release of damage-associated molecular patterns (DAMPs) from dying cells in the ischemic region [31,32]. These DAMP signals are detected by immune cells [33] and fibroblasts [34] to trigger an initial inflammatory and fibrotic response.

While the inflammatory response aids in clearing out dead cells and debris, the fibrotic cascade helps maintain tissue integrity through the deposition of matrix proteins such as collagen, fibronectin and tenascin C. Interestingly, deposition of this initial fibrotic tissue composition is imperative for regeneration [12]. Collagen and tenascin C are required for ventricular wall integrity [12], while fibronectin is necessary for the migration of new CMs to the infarct zone [35,36].

Intriguingly, while even adult human hearts have a small basal level of cell-cycling CMs [37,38], these cells are not sufficient to replace the lost myocardium after an MI. Thus, an injury-induced promotion of CMs to dedifferentiate and re-enter the cell cycle is a major pro-regenerative milestone [39]. In zebrafish, CM proliferation peaks between 7–14 days post-amputation (dpa), correlating with the time by which the fibrotic scar begins its regression [7]. By 30 dpa, the scar has largely resolved, having been replaced by new myocardium.

In the pro-fibrotic response, the lack of new CM synthesis leads to the formation of a more stable permanent scar by increasing its collagen deposition and crosslinking [40]. Over time, both the scar and the peri-infarct zone undergo further pathological remodeling, likely as a reactive process to the mechanical strain and stress the tissues are working under [40,41]. In the scar, muscle thinning is prevalent, causing the ventricle to dilate and leaving it vulnerable to rupture. In the peri-infarct zone, CMs grow in size to compensate for the increased workload and decreased ventricular wall tension. Unfortunately, these hypertrophic cells are also at high risk of apoptosis, which could in turn, initiate another wave of inflammation and fibrosis [40,41]. This perpetual cycling and remodeling may ultimately lead to heart failure.

## 3. Cardiac-Injury Response Modulators

Recovery of heart function following acute or chronic damage is the ultimate goal in cardiac regenerative medicine. Studies in the zebrafish and salamander have been key to identifying critical cellular processes and genetic circuits that enable these animals to naturally regenerate heart muscle. These circuits are the endogenous, default repair machinery that are unleashed upon injury, irrespective of organism age [42]. It is appreciated that the machinery that drives heart regeneration in these lower vertebrates are also highly conserved in adult mammals [43]. Thus, leveraging our growing knowledge of how the heart efficiently regenerates muscle and restores heart function will be key to unlocking the regenerative potential in humans.

### 3.1. Immune Cells

Over the past four years, much attention has been given to the immune response that immediately follows cardiac injury [10,20,22,25,26,44,45,46,47,48,49,50,51,52,53,54,55,56]. From these investigations, it is becoming clear that immune cells can greatly influence the course of the core cardiac repair program, controlling the balance between pro-fibrosis and pro-regeneration. Macrophages and regulatory T cells (Tregs) are two prominent immune cells that have emerged as central players during heart regeneration. Functional studies from numerous groups have shown CM proliferation is suppressed under conditions of cell depletion [49,53,55]. However, the mechanism of action exerted on CMs and the potential influence on other immune cells are outstanding questions within the cardiac regeneration discipline.

#### 3.1.1. Macrophages

Macrophages are remarkably unparalleled in their ability to influence the cardiac repair program through regulation and polarization of cytokine activity. Historically, macrophages are classified in two binary states of activity, the pro-inflammatory M1, and highly pro-regenerative and anti-inflammatory M2. M1 macrophages rapidly invade injury sites but the M2 subclass stimulates expression and release of growth factors that control matrix remodeling and angiogenesis while concurrently suppressing secondary inflammatory induced damage [57,58,59]. However, more recent findings indicate that macrophages are highly plastic, existing in shades of gray in a polarized state between the bookends of the M1 and M2 states [60,61,62]. Not surprisingly, this dynamic ability to adopt different activity states positions macrophages as central players in several cellular processes.

Outside their prominent role in removing cellular and tissue debris in and around the injured region [23], macrophages are bestowed the honor of mediating progression of the cardiac repair program. For example, while rapid accumulation of neutrophils is important for mounting the initial inflammatory response [63], their clearance by macrophage-mediated phagocytosis is imperative to prevent a prolonged inflammatory phase [50]. Likewise, macrophages are also important mediators of cardiac fibrosis. During the initial fibrotic response, macrophages contribute to scar formation by directly releasing matrix proteins as well as by promoting proliferation and activation of fibroblast through the release of stimulatory cytokines [23]. Later, to allow for scar regression, macrophages shut off the pro-fibrotic response by inactivating fibroblasts [10], and release MMPs to break down the extracellular matrix [61]. Thus, macrophages are key to initiation and resolution of the pro-fibrotic response in the context of tissue injury.

Unfortunately, the answer as to whether macrophages promote injury-induced proliferation of CMs is currently unresolved [10,44,46,50]. While two independent studies in the zebrafish [46,50] have reported a suppression of CM proliferation following clodronate liposome depletion of macrophages one day prior to cardiac injury, cardiac injuries using the same technique in the axolotl [10] and neonate mouse [44] have reported no differences in CM proliferation. Although all four reports revealed an otherwise similar impaired regenerative response, it is important to note that clodronate liposomes could directly affect other immune cells in a species-specific manner. Given the complexity and heterogeneity of macrophage subpopulations, and extensive intercellular communication between immune cells, assigning specific roles for macrophages during heart injury will likely require the use of more finely designed genetic tools to deplete subgroups of macrophages and more detailed cellular analyses.

#### 3.1.2. Regulatory T Cells (Tregs)

Intriguingly, while macrophages have substantial influential power over the cardiac repair program, they are not the only immune cell type endowed with this ability. Like macrophages, Tregs (CD4+ CD25+ FOXP3+) mediate various events in the cardiac repair program—playing favor to the pro-regenerative path. For example, Tregs are responsible for limiting inflammation [54,55,56]. Depletion of Tregs results in a massive influx of inflammatory cells in the infarct zone [55,56]. Additionally, while the presence of Tregs promotes the polarization of macrophages towards an anti-inflammatory M2-phenotype that function to resolve inflammation and repair the damaged tissue, their absence leads towards an enhanced inflammatory M1-phenotype [56]. A proper biphasic balance between these macrophage activation states is essential for performing their timely functions and thus preventing chronic inflammation (prolonged excessive M1) or fibrosis (early excessive M2) [24,52,64].

Given that macrophages are under the control of Tregs, it is not surprising that Tregs are mediators of fibrosis as well. Loss of Tregs results in sparse collagen deposition, creating a weak scar that is easily prone to rupture [55]. Alternatively, enhanced activation of Tregs increases collagen deposition, effectively accelerating scar formation [56].

By far, the most provocative role of Tregs lies within their compelling ability to stimulate injury-induced CM proliferation [49,53,55]. Unlike macrophages, this function has been shown to occur in vivo in the zebrafish [49], neonatal mouse [53] and adult mouse [55] as well as in human CMs co-cultured with Treg cells [53]. Currently, the only discrepancy between these three reports lies in regard to which Treg secreted factor(s) is/are responsible for stimulating CM proliferation. Zacchigna et al. [55] reported a set of six factors (Cst7, Tnfsf11, IL-33, Fgl2, Matn2, and Igf2) that individually, was capable of stimulating proliferation in cultured neonatal rat CMs. Importantly, viral-mediated delivery of these factors into the peri-infarct area of mouse hearts post-MI stimulated improvement in ejection fraction, increased EdU+CMs and reduced fibrosis [55]. Given that homologs for zebrafish Cst7, IL-33 and Matn2 have yet to be identified, it remains to be seen if the Zacchigna factors have conserved function between adult zebrafish and rodents.

By contrast, both Li et al. [53] and Hui et al. [49] were able to identify a single, yet different (amphiregulin, Areg [53]; neuroregulin-1, Nrg1 [49]) Treg cytokine that was able to stimulate heart regeneration. Since both of these cytokines are ligands for receptors in the erbB family [65], it is possible that the same downstream transduction cascade is being activated in neonatal mice with Areg [53] and in zebrafish with Nrg1 [49]. It is intriguing to postulate that these two cytokines may be interchangeable, as Nrg1 has been shown to induce CM proliferation in adult [66] and neonatal mice [67]. Additionally, as Areg is upregulated in zebrafish tissues upon injury, it would be interesting to determine if Areg could similarly stimulate CM proliferation in Treg-depleted zebrafish hearts. The interchangeability of these two stimulatory cytokines could further be tested by depleting one and testing if the addition of the other is sufficient for a regenerative response after cardiac injury.

#### 3.1.3. Other Immune Cell Types

While macrophages and Tregs have been recent focal points for studies in immunity and tissue repair, there is emerging evidence that other immune cells may also influence the cardiac repair program.

**B cells**: B lymphocytes, or B cells, have historically been studied in the context of adaptive immune responses, primarily through mechanistic investigations in antibody secretion [68]. More recent studies, however, suggest an important role in B cell function in coordinating the cardiac repair program. Zouggari et al. [69] reported that depletion of mature B cells, either genetically (deficiency in the Baff receptor) or through the use of an antibody against CD20 or Baff, lead to improved heart function in mice post-MI. Intriguingly, B cells with a deficiency in producing Ccl7 (also known as MCP-3) also exhibited this improvement, suggesting that a population of B cells that lack Ccl7 would be pro-regenerative [69]. The idea that a set of B cells could be pro-regenerative is reinforced by Goodchild et al. (2009), who found that a myocardial injection of bone marrow-derived B cells preserved cardiac function in rats post-MI by reducing CM apoptosis [70]. It would be interesting to determine if these bone marrow-derived B cells also lack Ccl7.

Interestingly, B cells have also been shown to polarize macrophages toward an M2-baised state both in co-culture and in vivo [71]. In this study, the B cell population was assessed as two subsets (B1, B2) with different tissue distributions and molecular properties. B1 cells colonize the gut lamina, peritoneal and pleural cavities, while the conventional B2 cells are found in the spleen and lymph nodes [72]. Molecularly, B1 cells are B220^1o^IgM^hi^CD11b^+^, whereas B2 cells are B220^hi^IgM^lo^CD11b^−^. Intriguingly, only the B1 subset was able to induce changes in the macrophage’s cytokine/chemokine profile, polarizing it toward an M2 state when under LPS stimulus [72]. These results support the idea that a subset of B cells, likely the B1 cells, is pro-regenerative. If so, this brings into question whether zebrafish B cells are more B1-like and whether cardiac function post-MI would be improved in mice with more B1 cells.

**Eosinophils**: Another immune cell that has risen in interest are the eosiphils, rare white blood cells associated with infection and asthma. A characteristic that separates eosinophils from most lymphocytes is the ability to store and rapidly secrete preformed cytokines [73]. Eosinophils release IL-4 and IL-13, which are common stimuli for polarizing macrophages toward in M2-state in culture [73,74] as well as modifying T-cell activity through the regulation of Notch signaling [75]. While direct studies connecting eosinophils with heart repair and regeneration remain outstanding, recent studies have shown that eosinophils synthesize and release factors such as TGFβ, VEGF, MMPs and nerve growth factors, signals shown to be critical in modulating the scarring vs. regeneration circuits of heart repair [76,77,78].

**Neutrophils**: Neutrophils are the first immune cell type recruited to the ischemic myocardium after injury [20]. Mice subjected to neutrophil depletion via intraperitoneal injections with a monoclonal antibody clone 1A8 exhibited increased fibrosis and progressively developed heart failure after an induced MI [48]. Surprisingly, while these mice had more M2-like macrophages, they had reduced expression of the phagocytosis receptor MerK that helps macrophages mediate clearance of apoptotic cells. Thus, while an early robust M2 macrophage state is unwanted, these results suggest that in the normal course of events when the neutrophils are cleared away, their absence likely influences the shift in an M1 dominant population to M2.

### 3.2. MicroRNAs (miRNAs)

The dynamic and intricate roles exerted by macrophage and Treg cells during heart regeneration are likely underscored by changes in gene expression of both cardiac and recruited immune cells to the injury site. Understanding how these signaling cascades are temporally and spatially confined is a critical objective in the quest to stimulate heart regeneration processes in humans. A key group of gene expression regulators are the small, non-coding RNAs termed miRNAs. These small (~19–22 nucleotide long) single-stranded noncoding RNAs regulate target genes by forming a RNA-induced silencing complex (RISC) with a member of the Argonaute (Ago) protein family [79]. Upon the miRNA base-pairing with the 3’ untranslated region of a target mRNA, protein translation is abrogated, either through transcript degradation or inhibition of translation [80]. A single miRNA commonly targets multiple genes, making miRNAs a powerful biological rheostat of developmental programs.

#### 3.2.1. MiRNA Regulation of Cardiomyocyte (CM) Proliferation and Fibrosis

MiRNA regulation of gene expression is seen in many biological and pathological processes [81], including those involved in the cardiac-injury repair program. In the past 9 years, 19 miRNAs have emerged as potent suppressors or stimulators of fibrosis or CM proliferation after heart injury (Table 1). Impressively, miR101a mediates both events. While early downregulation of miR-101a is required for CM proliferation [11], its later upregulation is required for scar removal [11,82,83]. Interestingly, these effects are driven in part by the control of c-fos expression within the epicardium and immune cells that penetrate the wounded apex. This dynamic modulation of miR-101 demonstrates how careful fine-tuning of the biological state is necessary for the progression of a pro-regenerative response. Mir-21 and miR-33 are two additional provocative miRNAs. In addition to regulation of fibrosis [84,85] (Table 1), miR-21 and miR-33 are hypothesized to attenuate inflammation by promoting macrophage polarization towards an M2 phenotype [86,87], possible through its induction of Tregs [87]. The extent that these and other miRNAs may influence the activity of other immune cells, however, remains to be defined.

#### 3.2.2. Non-Coding Databases

Our understanding of miRNAs in mediating the cardiac-injury response is progressively growing, and will only increase with the recent establishment of two comprehensive databases on non-coding RNAs (ncRNAs). The HDncRNA database allows for the identification of ncRNAs that are either predicted or known to be associated with heart disease—including MI [102]. While the database contains a wealth of information with the ability to search through six different mammalian species (human, mouse, rat, pig, calf and dog), it lacks information on regenerative models and is thus limited to identifying ncRNAs involved in the pro-fibrotic response. On the other hand, the RegenDbase contains genes, transcripts and ncRNA information from both non-regenerative (humans and adult mice) and regenerative heart models (neonatal mice, zebrafish and axolotls) [29]. Expression profiling from various time-points during zebrafish heart regeneration helps capture dynamic changes in the presence of ncRNAs, which can be important for understanding its suppressive or promoting role at different stages in the injury response. These resources and other emerging databases provide a foundation for performing comparative studies between highly regenerative model systems in an effort to elucidate a core genetic signature that underscores cardiac regenerative capacity.

## 4. Conclusions

The lack of therapeutic treatments to repair damaged cardiac tissue after a heart attack strongly limits the ability to improve cardiac function post-MI. The development of treatments targeted at modulating immune cells or the expression of miRNAs hold great promise in fulfilling this need. At the heart of this central goal stands the zebrafish, which has revolutionized our thinking about the regenerative potential of the adult mammalian heart. In just 15 years, work on the zebrafish has not only demonstrated that heart regeneration is possible, but it has also powered our understanding of the cellular processes and molecular factors that guide CM regeneration and scar tissue resolution following acute heart damage. Leveraging this knowledge to distill a core regenerative circuit is likely to be instrumental in unlocking the dormant regenerative potential in human hearts.

Chief among our emerging *knowledge* base is the recognition that immune cells play a key role in limiting pro-inflammatory and maximizing pro-regenerative cytokine activity. Locking the activity of immune cells to this “Goldilocks” zone may be a fundamental role for non-coding RNAs, especially miRNAs. Recognized as biological rheostats, miRNAs can quickly stimulate the shift from inflammation to regeneration by modulating expression of an individual or several miRNAs. Undoubtedly, decoding the regulatory control of miRNAs on immune cell activity will be a strong foundation for developing therapeutic strategies that maximize regenerative capacity and improve heart function after MI.

## Figures and Tables

**Table 1:** miRNA-mediated suppression (−)/promotion (+) of events in the cardiac-injury response.

miRNAs	Fibrosis		Cardiomyocyte (CM) Proliferation		References
miR-21	(+)	*pten* ^1^			[84,86]
	Promotes M2 polarization Stimulates IL-10 release				
miR-24	(−)	*furin*			[88]
miR-29	(−)	*eln, fbn1, col1α1, col1α2, col3α1*			[89]
miR-33	(+)	*mmp16*			[85,87]
	Suppresses M2 polarization Inhibits Treg induction				
miR-206	(−)	*timp3*			[90]
miR-223	(+)	*rasa1*			[91]
miR-328	(−)	*tgfβr3*			[92]
miR-370	(−)	*tgfβr2*			[93]
miR-433	(+)	*azin1, jnk1*			[94]
miR-101a	(−)	*fosab (c-fos), tgfβr1*	(−)	*fosab (c-fos)*	[11,82,83] ^2^
miR-15 family (miR-195)			(−)	*chek1*	[95,96]
miR-17-92 cluster			(+)	*pten*	[97]
miR-26a			(−)	*ezh2*	[28]
miR-34a			(−)	*bcl2, cyclin D1, sirt1*	[98]
miR-128			(−)	*suz12*	[99]
miR-133			(−)	*cx43*	[16]
miR-199a			(+)	*homer1, hopx, clic5*	[100]
miR-302-367			(+)	*mob1b, lats2, mst1*	[101]
miR-590			(+)	*homer1, hopx, clic5*	[100]

^1^ Gene target(s) of miRNA. ^2^ CM proliferation reference.
